# Mandibular Odontogenic Tumors That Caused Delayed Eruption of the First Molar: A Case Report of Two Patients

**DOI:** 10.7759/cureus.66214

**Published:** 2024-08-05

**Authors:** Hiroshi Shiratsuchi, Shunsuke Namaki, Takaaki Tamagawa, Machi Hosaka, Yoshiyuki Yonehara

**Affiliations:** 1 Department of Oral and Maxillofacial Surgery Ⅱ, Nihon University School of Dentistry, Tokyo, JPN

**Keywords:** first molar, ameloblastic fibro-dontoma, odontoma, permanent teeth impaction, oral and maxillofacial surgery

## Abstract

Delayed eruption of permanent teeth during the replacement period is relatively common in clinical practice; however, impaction of the mandibular first molar is rare. There are various causes of delayed eruption of permanent teeth such as odontogenic cysts and tumors. This article describes the management of two odontogenic tumors that caused the delayed eruption of the mandibular first molar. In Case 1, an eight-year-old boy was diagnosed with an unerupted right mandibular first and second molar that had an odontogenic tumor around them. Radiographic examination revealed well-defined unilocular radiolucency with impacted first and second molars and scattered radiographic opaque images at the right mandibular. The lesion was completely curettaged with extraction of the second molar, and the first molar was fenestrated. Pathological microscopic examination provided the diagnosis as an ameloblastic fibro-odontoma. In Case 2, an 11-year-old boy was diagnosed with an unerupted right mandibular first molar that had an odontogenic tumor around it. Radiographic examination revealed well-defined unilocular radiolucency with an impacted first molar and scattered radiographic opaque images at the right mandibular The lesion was completely curretaged, and the first molar was fenestrated. Pathological microscopic examination provided the diagnosis of odontoma. Among these two cases, the preserved first molar erupted at each regular position. We demonstrated that even if an odontogenic tumor is present along with an impacted molar, removal of the tumor can result in the eruption of the impacted tooth.

## Introduction

Delayed tooth eruption occurs when teeth emerge later than the average expected timeframe. Various factors can contribute to the delayed eruption of permanent teeth. Systemic factors encompass conditions such as cleidocranial dysplasia [1]. Local factors include the abnormal position of tooth germ, abnormal formation of teeth, late retention of deciduous teeth, presence of supernumerary teeth, and impact of odontogenic cysts and tumors [2]. According to previous case reports, the incidence of permanent teeth impaction ranges between 5.6% and 18.8% [3-7]. This is most prevalent in the upper and lower third molars, maxillary canines, and mandibular second premolars but rarely occurs in the first molars [3-7]. Additionally, the probability of the mandibular first molar becoming impacted is 0.01% [7]. Odontogenic tumors occur most frequently in young patients and are often accompanied by impacted teeth [2,8-10]. In the treatment of odontogenic tumors, the decision to preserve or remove the unerupted tooth depends on the patient’s clinical status [11]. This article describes the management of two odontogenic tumors that caused the delayed eruption of the mandibular first molar. Even if an odontogenic tumor is present along with an impacted first molar, removal of the tumor can result in the eruption of the impacted tooth.

## Case presentation

Case 1

**Figure 1:**
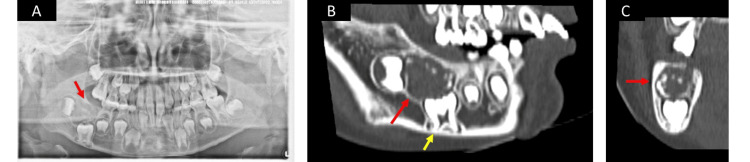
Panoramic radiograph and CBCT of case 1 (at the initial visit) (A) A panoramic radiograph obtained during the initial visit showing a well-defined unilocular radiolucency (red arrow) with impacted first and second molars at the right mandibular area; (B, C) A dental cone beam computed tomography image obtained at the initial visit showed a well-defined unilocular radiolucency (red arrow) with impacted first and second molars and scattered radiographic opaque images at the right mandibular area. Morphological abnormalities in the tooth root were observed at the impacted first molar (yellow arrow).

An eight-year-old Japanese boy was initially examined by a dentist due to the delayed eruption of his first molar. He was subsequently referred to the Oral and Maxillofacial Surgery Department of Nihon University Dental Hospital. Initial examination revealed impaction of the lower right first and second molars and a bony swelling on the buccal side. No complaints of spontaneous or evoked pain were noted in the right mandibular area. A panoramic radiographic examination indicated a well-defined unilocular radiolucency with impacted first and second molars at the right mandibular (Figure 1A). Dental cone beam computed tomography (CBCT) examination revealed a well-defined unilocular radiolucency measuring 19.5×14.5 mm with impacted first and second molars and scattered radiographic opaque images at the right mandibular (Figures 1B, 1C). Additionally, morphological abnormalities in the tooth root were observed at the impacted first molar (Figure 1B).

**Figure 2:**
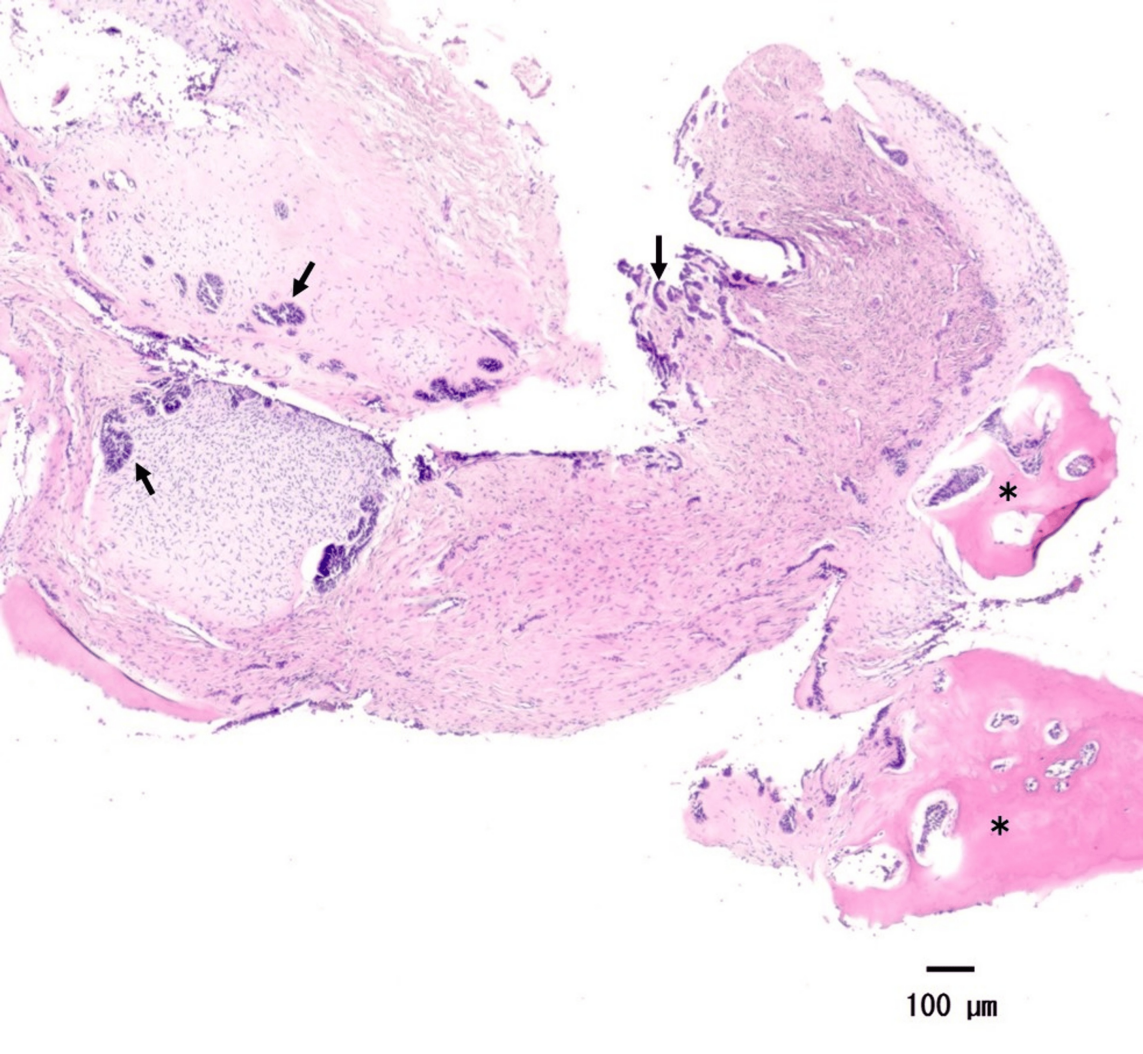
Histological findings of the surgery specimen (Case 1) Hematoxylin and eosin-stained section revealing the formation of a dentin-like tissue (*) and the proliferation of island-like and cord-like odontogenic epithelium (black arrows)

**Figure 3:**
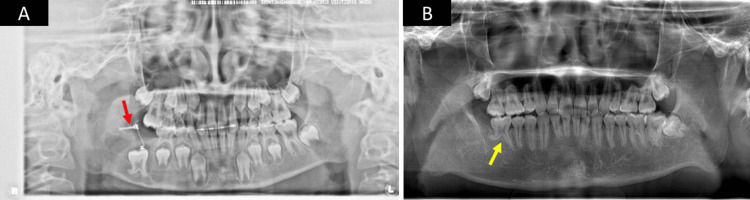
Postoperative panoramic radiograph of Case 1 (A) A panoramic radiograph obtained eight months postoperatively showing bone regeneration, an anchoring device placed at the crown of the first molar, and anchor screw implantation (red arrow) at the right mandibular area; (B) A panoramic radiograph obtained 10 years postoperatively showing regenerated bone at the right mandibular area. The first molar, which still had morphological abnormalities in the tooth root, exhibited a normal direction of eruption (yellow arrow).

Based on the imaging and clinical findings, a clinical diagnosis of the lesion was considered to be odontoma, ameloblastic fibro-odontoma, and calcifying epithelial odontogenic tumor. A biopsy was performed, which confirmed the pathological diagnosis of odontoma (complex type). After the biopsy, the lesion was completely excised through curettage under general anesthesia. The second molar was extracted due to its misalignment, the first molar was preserved in anticipation of eruption, and the left wound remained open. Pathological microscopic examination revealed the formation of dentin-like tissue and the proliferation of island-like and cord-like odontogenic epithelium. Based on these results, the lesion was diagnosed as ameloblastic fibro-odontoma (AFO, developing odontoma) (Figure 2). After eight months of follow-up, regenerated bone was observed. Hence, an anchor screw was implanted at the right mandibular area, and an anchoring device was placed on the crown of the impacted first molar (Figure 3A). We performed traction on the first molar along with the orthodontist who worked in the patient's referring dental clinic. The duration of the traction was about five months. A panoramic radiograph taken after a 10-year follow-up showed regenerated bone at the right mandibular area. The preserved first molar, which still had morphological abnormalities in the tooth root, exhibited a normal direction of eruption (Figure 3B).

Case 2

**Figure 4:**
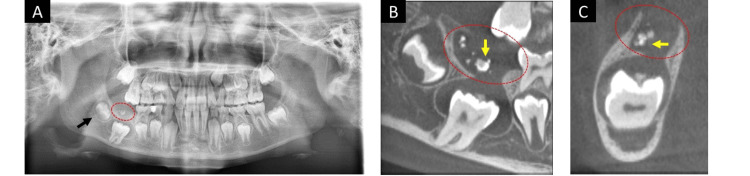
Panoramic radiograph and CBCT of Case 2 (at the initial visit) (A) Panoramic radiograph obtained during the initial visit showing a well-defined unilocular radiolucency (dashed red circle) with impacted first and second molars at the right mandibular area. At the right mandibular second molar, tooth crown development was slower than the left one (black arrow); (B, C) A dental cone beam computed tomography image obtained during the initial visit showing a well-defined unilocular radiolucency (dashed red circle) with impacted first molar and scattered radiographic opaque images (yellow arrow) at the right mandibular area

An 11-year-old Japanese boy was initially examined by a dentist due to the delay in the eruption of the first molar. He was subsequently referred to the Oral and Maxillofacial Surgery Department of Nihon University Dental Hospital. Initial examination revealed an impaction of the lower right first molar, with no signs of swelling on the buccal side of the alveolar region. Moreover, no complaints of spontaneous or evoked pain in the right mandibular area were reported. Panoramic radiography indicated a well-defined unilocular radiolucency with impacted first and second molars at the right mandibular area (Figure 4A). At the right mandibular second molar, tooth crown development was slower than the left one (Figure 4A). Dental CBCT examination revealed a well-defined unilocular radiolucency measuring 15.5×7.0 mm with an impacted first molar and scattered radiographic opaque images at the right mandibular area (Figures 4B, 4C).

**Figure 5:**
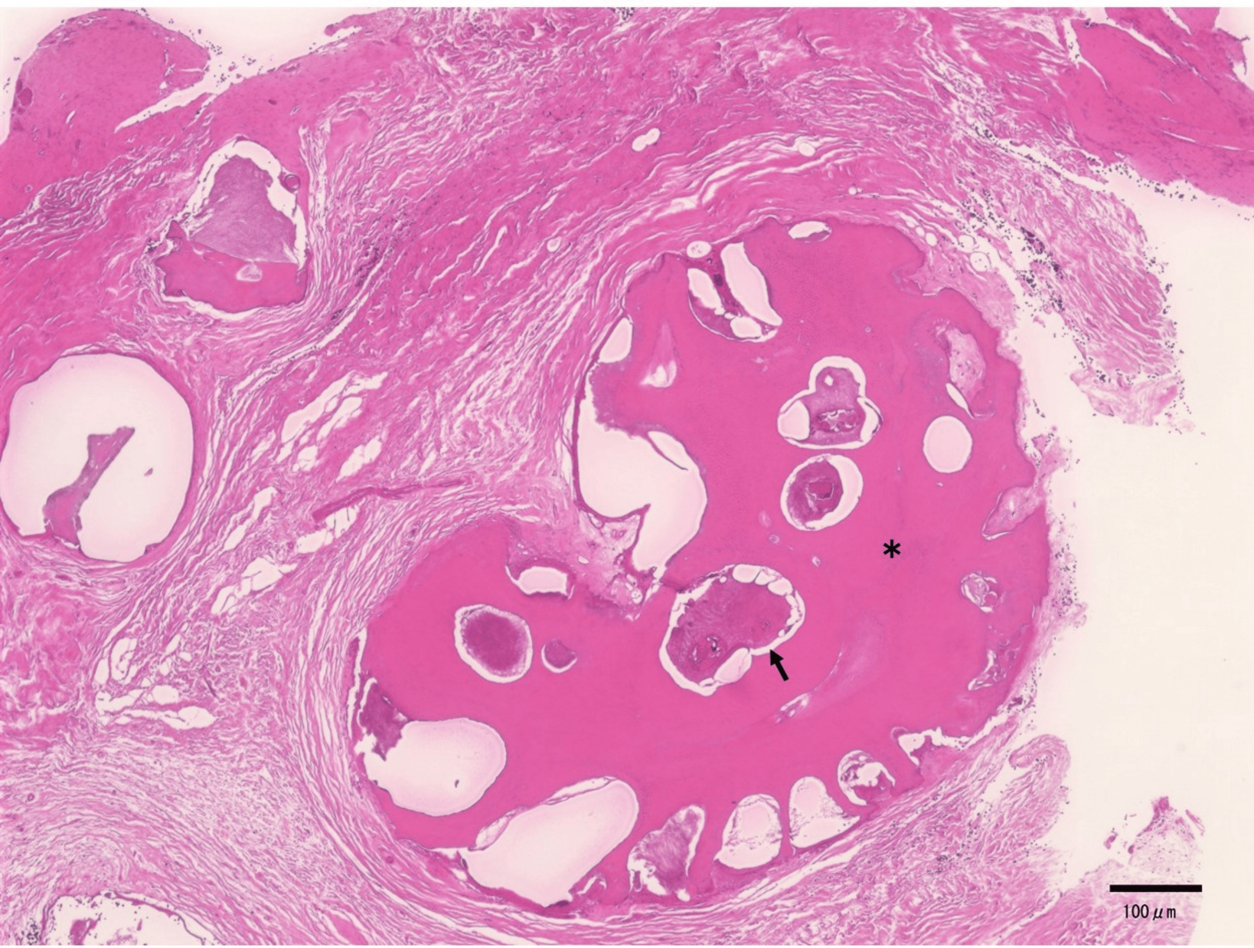
Histological findings of the surgery specimen (Case 2) Hematoxylin and eosin-stained section revealing the formation of a dentin-like tissue (*) and a tissue resembling dental pulp (black arrow)

**Figure 6:**
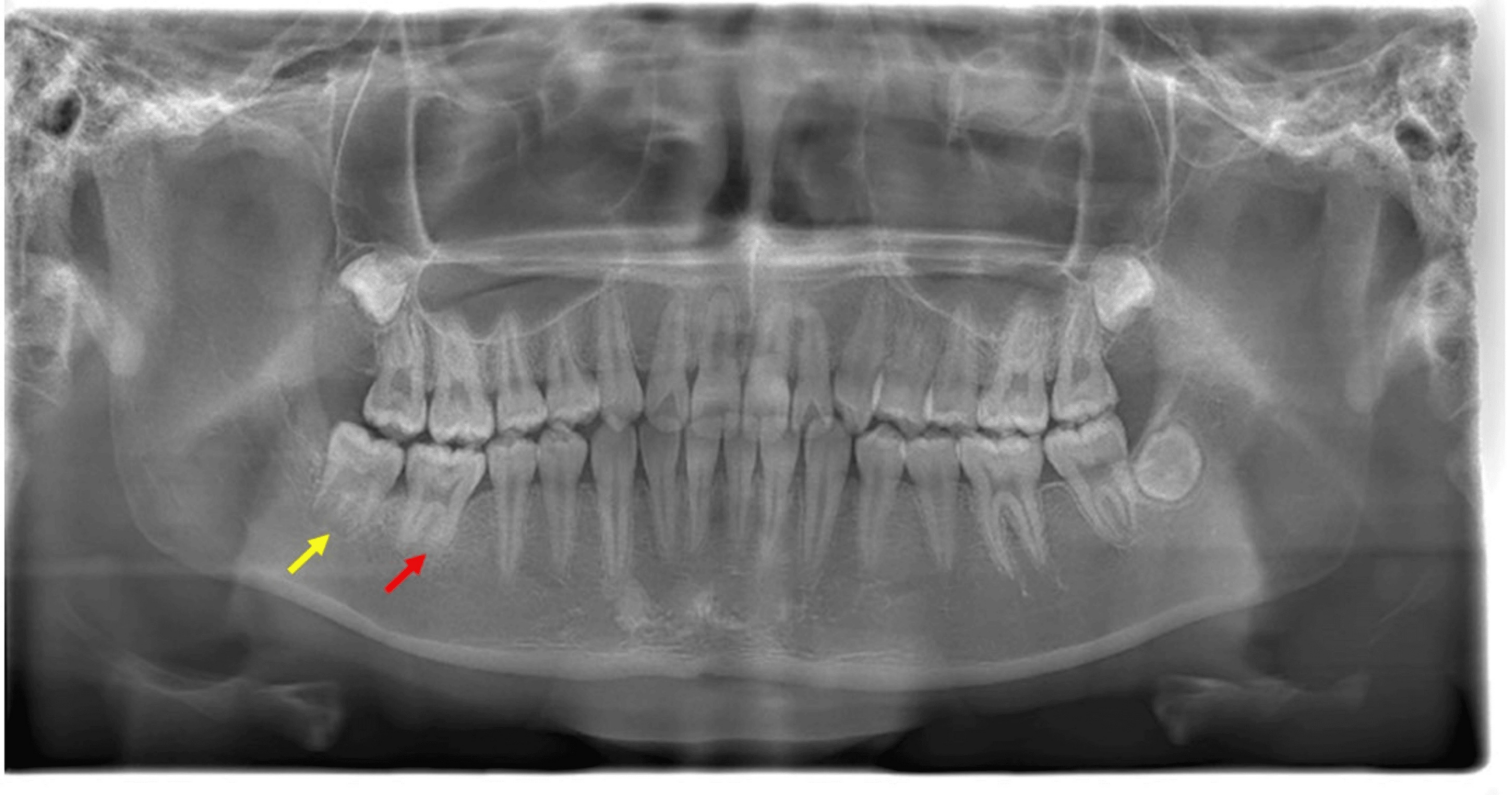
Postoperative panoramic radiograph of Case 2 A panoramic radiograph obtained four years postoperatively showing bone formation at the right mandibular area while the first molar demonstrated a normal direction of eruption (red arrow). The second molar apical closure was not obtained, however, a normal direction of eruption was shown (yellow arrow).

In accordance with the imaging and clinical findings, a clinical diagnosis of the lesion was considered to be ameloblastic fibro-odontoma, calcifying epithelial odontogenic tumor, or calcifying odontogenic cyst. The lesion was completely excised via curettage under general anesthesia. The first molar was preserved in anticipation of eruption, and the left wound remained open. Pathological microscopic examination revealed the formation of a dentin-like tissue and a tissue resembling a dental pulp. Based on this result, the lesion was diagnosed as odontoma (complex type) (Figure 5). A panoramic radiograph taken after four years of follow-up showed regenerated bone at the right mandibular area. The preserved first molar and second molar exhibited a normal direction of eruption. However, the second molar apical closure was not obtained (Figure 6).

## Discussion

Delayed tooth eruption occurs when the teeth emerge later than the average timeframe. Various factors can cause delays in the eruption of permanent teeth. Systemic factors include conditions such as cleidocranial dysplasia [1]. Local factors include the abnormal position of tooth germ, abnormal formation of teeth, late retention of deciduous teeth, presence of supernumerary teeth, and impact of odontogenic cysts and tumors [2]. Baden reported that the delayed eruption of permanent teeth is frequently observed in maxillary canines [3]. Previous case reports suggested that permanent tooth impaction commonly occurred in the upper and lower wisdom teeth, maxillary canines, and mandibular second premolars but rarely in the mandibular first molars [4-7]. In this case report, the mandibular first molar was impacted due to the presence of an odontogenic tumor (Case 1: AFO; Case 2: odontoma, complex type).

In the 2005 World Health Organization (WHO) classification, AFO is described as a tumor characterized by the proliferation of both odontogenic epithelium and mesenchymal tissue. It is regarded as a mixed odontogenic tumor that leads to the formation of dentin and enamel [8]. In the 2017 WHO classification, AFO was defined as a developing odontoma [9]. In a previous statistical study of AFO, Buchner noted that this tumor occurs most frequently in the mandibular molar region [10]. The average age of onset of AFO is approximately 10 years, with the condition being most common in individuals up to age 20 years [11]. Among odontogenic tumors, AFO is relatively rare, occurring in less than 1% [8]. Additionally, AFO typically presents with fewer clinical symptoms. It is painless and develops slowly. It is often accompanied by tooth impaction and associated with delayed tooth eruption [11]. Radiologically, AFO is characterized by a well-demarcated radiolucent image with opaque areas and is frequently accompanied by tooth impaction [11]. In our case report, both patients exhibited a well-demarcated radiolucent image in the mandibular molar region with a mixed opaque image and tooth impaction. Surgical removal is the first-line treatment for AFO owing to its solid and encapsulated nature [12]. The decision to preserve or remove the unerupted tooth depends on the patient’s clinical status [11]. In our case, the tumor border was well-defined. Hence, an excision was performed, and the incision was left open to facilitate the eruption of the impacted first molar. However, the impacted second molar was misaligned. Therefore, the tooth was considered difficult to erupt. Thus, it was extracted. Postoperative orthodontic treatment guided the impacted first molar to the correct position.

Odontomas are mixed epithelial and mesenchymal tumor-like malformations (hamartomas) composed of dental hard and soft tissues [13]. They are subdivided into compound odontoma and complex odontoma [13]. Odontomas are the most common odontogenic tumors [13]. They are typically diagnosed during the first two decades of life and exhibit no sex predilection [13]. Although odontomas can occur in any tooth-bearing area, compound odontomas are primarily located in the anterior maxilla while complex odontomas are commonly found in the posterior mandible, followed closely by the anterior maxilla [13,14]. Kaugars reported that the incidence of odontomas in the molar region gradually increased with each successive decade of life [14]. In our case, the presence of an odontoma was associated with the impacted right mandibular first molar. Odontomas are removed by conservative surgery due to their low growth potential [13-15]. Recurrence after complete removal is unusual; however, Tomizawa reported a case of recurrence [2]. Morning et al. reported that in 17 out of 35 patients who developed odontomas with impacted teeth, the removal of odontoma allowed the impacted teeth to erupt [15]. In our case, an open incision was made to promote the eruption of the impacted first molar. Four years after surgery, the preserved first molar had erupted in a direction that would facilitate a proper occlusal relationship. The second molar apical closure was not obtained; however, a normal direction of eruption was shown. The reason why the second molar had an incomplete root is thought to be that the preoperative crown formation was delayed compared to the opposite side.

Researchers have extensively discussed the relationship between AFO and odontoma [16-18]. AFO and odontoma share many common clinical symptoms and radiographic findings. Histopathologically, complex odontomas in the developing stage have immature hard tissues and ectodermal mesenchyme resembling the enamel organ and dental papilla. They are considered similar to AFO [16-18]. Slootweg stated that AFO may progress into a complex odontoma, suggesting that they are the same type of lesion [17]. Additionally, Gardner reported distinguishing between AFO and developing odontoma is challenging due to their histopathological similarities [18]. Based on these reports, AFO was defined as a developing complex odontoma in the 2017 WHO classification. Conservative surgical removal of AFO and odontoma is generally considered the first-line treatment, with a favorable prognosis [2,8-10,13,14]. However, previous case reports documented the malignant transformation of AFO into ameloblastic fibrosarcoma. Therefore, careful follow-up is required [19,20].

## Conclusions

We present the cases of two patients who developed odontogenic tumors in the mandible that caused the delayed eruption of the first molar. A detailed discussion of these cases was provided along with a literature review. In Case 1, an orthodontic approach was performed for impacted mandibular first molar eruption after surgery. In Case 2, a natural eruption was shown at the mandibular first molar after surgery. In both cases, the mandibular first molar was compressed inferiorly by the tumor, but because it was vertically impacted, the eruption could be stimulated by the tumor removal. Even if an odontogenic tumor is present along with an impacted molar, removal of the tumor can result in the eruption of the impacted tooth. In Case 1, the mandibular second molar was severely displaced and horizontally impacted, so it was extracted, but in Case 2, the mandibular second molar was only slightly displaced and slightly compressed downward, so it was preserved and naturally erupted. Thus, the decision as to whether or not to preserve an impacted tooth should be based on the degree of its displacement.
